# Complete Blood Count (CBC) Parameters as a Cost-Effective Tool for Early Diagnosis of Pediatric Sepsis: A Retrospective Cross-Sectional Study

**DOI:** 10.7759/cureus.89661

**Published:** 2025-08-08

**Authors:** Namita Mishra, Ashvini Rana, Pankaj Kumar, Rasna Gupta, Mritunjay Kumar

**Affiliations:** 1 Department of Paediatrics, All India Institute of Medical Sciences, Raebareli, Raebareli, IND; 2 Department of Pathology, All India Institute of Medical Sciences, Raebareli, Raebareli, IND

**Keywords:** cbc parameters, complete blood count, cost effective analysis, early identification and diagnosis, low-resource setting, pediatrics, sepsis, timely intervention

## Abstract

Introduction: Early recognition of pediatric sepsis is crucial for timely intervention, prevention of mortality, and improving long-term outcomes in children. However, the lack of advanced diagnostics in resource-limited settings poses a significant challenge to early diagnosis and intervention. Complete blood count (CBC) parameters are routinely performed, cost-effective, and readily available, yet their diagnostic utility in pediatric sepsis remains underutilized. This study aims to explore the utility of CBC markers for the early diagnosis of pediatric sepsis, focusing on a cost-effective analysis that is suitable for routine application in low-resource settings.

Methods: This retrospective cross-sectional case-control study was conducted in the Pediatric Intensive Care Unit (PICU) of a tertiary care center in Raebareli, India, between July 2021 and March 2024. A total of 200 pediatric patients (100 with sepsis and 100 controls) aged two months to 15 years were included. Sepsis was diagnosed based on the recently developed Phoenix Sepsis Score (≥2). Medical records were reviewed to extract CBC parameters, including hemoglobin, total leukocyte count, absolute neutrophil count, lymphocyte count, platelet count, red cell distribution width (RDW), and mean platelet volume (MPV). Derived indices such as neutrophil-to-lymphocyte ratio (NLR), platelet-to-lymphocyte ratio (PLR), and platelet mass index (PMI) were calculated. Statistical tests included the Mann-Whitney U test for non-categorical data and Fisher's exact test for categorical data. Receiver operating characteristic (ROC) curve analysis was performed to evaluate the diagnostic accuracy of the significant CBC parameters and indices. A p-value <0.05 was considered statistically significant.

Results: The median age of the population was 4.7 (1.0-7.75) years. Sepsis patients had a significantly longer hospital stay (p = 0.008), reflecting increased healthcare burden in children with sepsis. CBC parameters and indices that showed significant association with pediatric sepsis included hemoglobin (p=0.01), platelet count (p=0.001), RDW (p=0.037), NLR (p=0.028), PLR (p=0.031), and PMI (p<0.01). MPV and platelet distribution width (PDW) did not show significant differences. The ROC curve analysis indicated that PMI had the best diagnostic accuracy, as suggested by the highest area under the curve (AUC) (0.61). However, none of these test variables emerged as a standalone parameter for the diagnosis and need to be reviewed in combination with other parameters and indices.

Conclusion: The CBC parameters - such as hemoglobin levels, platelet count, RDW, and indices like the NLR, PLR, and PMI - showed significant differences in children with sepsis. Although the AUC of none of these parameters approached 0.7, however, CBC parameters may offer a practical, low-cost adjunct for supporting early suspicion of pediatric sepsis in resource-limited settings. While in the current study, no single marker demonstrated sufficient accuracy as a standalone diagnostic marker, combining multiple parameters may improve early recognition and guide timely intervention. Further adequately powered prospective studies taking into account the confounding factors are required to validate the role of these markers and indices in the early diagnosis of pediatric sepsis.

## Introduction

Sepsis is a major global cause of pediatric morbidity and mortality, particularly in low- and middle-income countries (LMICs). The Global Burden of Disease Study (2017) estimated 48.9 million sepsis cases and 11 million deaths globally, with over 20 million cases in children under five years [1]. A systematic review by Fleischmann-Struzek et al. reported a global pediatric sepsis incidence of 1.2 million annually, though likely underestimated in LMICs [2]. The Sepsis Prevalence, Outcomes, and Therapies (SPROUT) Study reported a sepsis prevalence of 8.2%, with wide intercontinental variation - from 6.2% in Europe to 23.1% in Africa (p < 0.001) - and a 25% mortality rate across Pediatric Intensive Care Units (PICUs) worldwide [3]. Mortality remains significantly higher in resource-limited settings, where guideline adherence is lower, highlighting the need for early recognition and intervention [4].

Children with sepsis often require intensive support, including vasopressors, mechanical ventilation, and prolonged hospitalization, leading to substantial health resource utilization [4]. Importantly, the long-term consequences are often detrimental. Studies like Life After Pediatric Sepsis Evaluation (LAPSE) have demonstrated that up to 47% of pediatric sepsis survivors experience moderate to severe functional limitations at discharge, with 20% experiencing a decline in health-related quality of life persisting beyond one year [5]. In India, 51% of patients had worsened physical and 28% had cognitive dysfunction at discharge [6].

Early and accurate diagnosis of pediatric sepsis remains a challenge due to its nonspecific clinical manifestations and the limited reliability of currently available biomarkers. Conventional biomarkers, such as procalcitonin (PCT), C-reactive protein (CRP), and lactate, have been widely studied; however, none offer sufficient sensitivity or specificity to serve as standalone diagnostic tools [7,8]. Advanced biomarkers such as interleukins, CD64, presepsin, and genomic assays are expensive and not routinely available in most clinical settings [9,10]. Their reliance on specialized technologies, such as flow cytometry, enzyme-linked immunosorbent assay (ELISA), or polymerase chain reaction (PCR), limits their practicality, particularly in low-resource settings where infrastructure and technical expertise may be lacking.

The 2005 International Pediatric Sepsis Consensus Conference provided pediatric-specific systemic inflammatory response syndrome (SIRS) criteria for sepsis diagnosis, emphasizing the need to account for age-specific physiological parameters [11]. However, their utility has been questioned due to poor discrimination and overdiagnosis, prompting calls for better tools, especially in LMICs. While tools such as the Pediatric Sequential Organ Failure Assessment (pSOFA) and Pediatric Logistic Organ Dysfunction (PELOD) scores are helpful, their complexity limits bedside applicability in resource-limited settings [12,13]. Considering these loopholes, this research was conducted to study the role of CBC parameters in the early diagnosis of sepsis in children.

Complete blood count (CBC) parameters; white blood cell (WBC), absolute neutrophil count (ANC), absolute lymphocyte count (ALC), platelet counts, hemoglobin, red cell distribution width (RDW), mean platelet volume (MPV), platelet distribution width (PDW), along with derived indices such as neutrophil-to-lymphocyte ratio (NLR), platelet-to-lymphocyte ratio (PLR), and platelet mass index (PMI), have shown considerable promise as accessible, affordable, and rapid markers of systemic inflammation. Neutrophils and lymphocytes are key immune cells in defending against infection. In sepsis, white blood cell counts vary depending on the individual's immune status, the type of infection, and the stage of the disease [14]. Typically, neutrophilia and lymphopenia indicate systemic infection. Thrombocytopenia is another hallmark of critical illness; in sepsis, it results from hemodilution, increased consumption, immune destruction, and splenic sequestration [15]. In response, the bone marrow releases larger, younger platelets into circulation, reflected in elevated MPV and PMI [16]. Preliminary studies suggest that certain CBC parameters may aid in early sepsis identification, but data in pediatric populations, particularly from Indian PICUs, remain sparse.

Given the urgent need for cost-effective diagnostic tools suitable for LMICs, this study aims to evaluate diagnostic accuracy and identify significant predictive CBC markers in early sepsis diagnosis in a pediatric intensive care unit.

## Materials and methods

Study design and setting

A case-control study was conducted in the PICU at the Department of Pediatrics in a tertiary care center located in Raebareli district, Uttar Pradesh (U.P.), India, from July 1, 2021, to March 31, 2024.

Sample size and sampling

The data of patients admitted to the PICU for various critical illnesses since July 2021 were extracted from electronic and written medical records. Given the retrospective nature of the study and resource constraints, a convenience sample of 100 patients per group was selected. This number was considered sufficient to provide preliminary evidence of differences in CBC parameters between children with and without sepsis, with acceptable power to detect moderate effect sizes. This sample size strikes a balance between practical constraints and statistical reliability. A non-probability consecutive sampling method was used for the selection of participants. Patients with a PICU stay of less than 24 hours and those who expired on the first day of admission were excluded. In addition, patients with known chronic systemic illness, including tuberculosis, cancer, liver disease, renal disease, congenital heart disease, inflammatory bowel disease, celiac disease, and immunodeficiency conditions, were excluded. Children who had received antibiotics prior to admission were also excluded.

Data collection and procedure

Patient diagnosis, age, gender, and hospital registration number, along with CBC parameters and CRP, were documented. Blood and urine cultures were recorded. In the CBC, the following parameters were recorded: hemoglobin, white blood cells, ANC, ALC, platelet count, RDW, and MPV. The ratio of absolute neutrophil count to absolute lymphocyte count, as well as platelet count to absolute lymphocyte count, was calculated as NLR and PLR, respectively, along with PMI as a product of platelet count and MPV. Blood cultures were sent in all patients in both groups. Need for blood transfusions, inotropic drugs, noninvasive or invasive mechanical ventilation, and patient outcome were also recorded. The participants for whom any of the required data was missing were not included in the study. Since all the data were obtained retrospectively from the hospital information system, informed patient consent was not taken. Approval for the study was obtained from the institutional ethics committee (2024-4-OTH-Exp-7).

The recently validated Phoenix Sepsis Score (PSS) was used to identify patients with sepsis [17]. PSS provides a contemporary, age-specific, organ dysfunction-based approach to diagnosing pediatric sepsis. This tool, unlike earlier SIRS-based definitions, aligns with the evolving understanding of sepsis as a dysregulated host response causing life-threatening organ dysfunction. The Phoenix Sepsis Score encompasses cardiovascular, respiratory, neurological, and coagulation components, facilitating more accurate risk stratification and the early identification of critically ill children.

In PSS, a score is provided based on Glasgow Coma Scale, lactate levels, need for vasopressors, need for respiratory support, PaO2, SpO2, FiO2, mean arterial pressure, platelet count, and coagulation profile. Individual score was calculated for each parameter and were summed together to get the final score. Children with suspected infection and a PSS score >2 were considered to be septic. An equal number of patients with a PSS of <2, admitted to the PICU and ward, were randomly selected as controls.

Statistical analysis

The collected data were entered into Microsoft Excel (Redmond, WA, USA) and analyzed using SPSS version 30.0 (IBM Corp., Armonk, NY, USA). The Kolmogorov-Smirnov and Shapiro-Wilk tests were used to check the homogeneity and normality of the dataset. All continuous data were represented as medians and interquartile ranges (IQRs). A percentage was used to represent the categorical data. To compare medians, the Mann-Whitney U test was employed. To compare the categorized results, Fisher's exact test was applied. A p-value <0.05 was considered statistically significant. The receiver operating characteristic (ROC) curve was used to analyze the diagnostic values of significant CBC parameters and ratios. Sensitivity, specificity, positive predictive value, negative predictive value, positive likelihood ratio, and negative likelihood ratio were calculated for each parameter and ratio.

## Results

**Figure 1:**
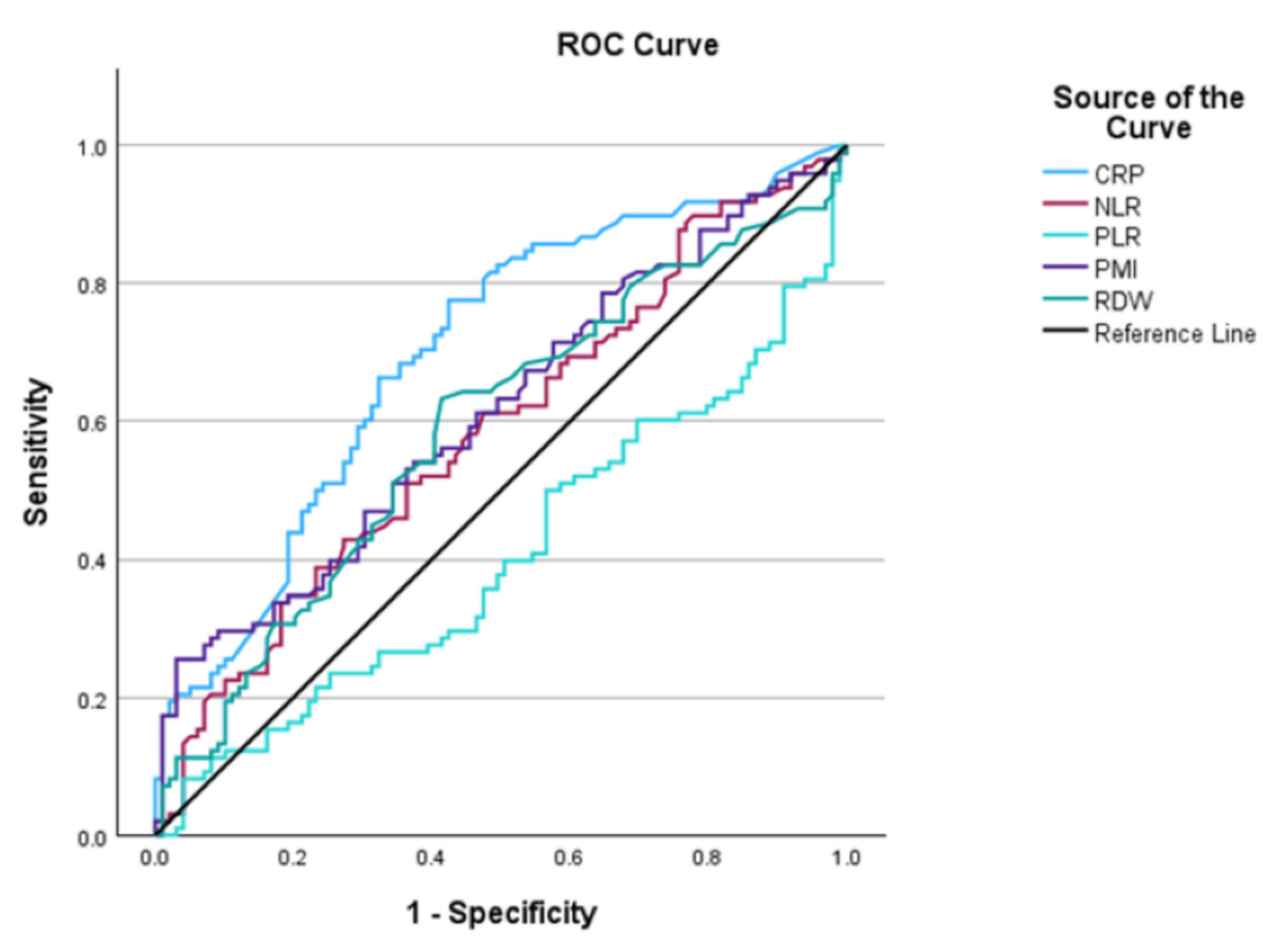
ROC Analysis of Test Parameters CRP: C-reactive protein (AUC-0.7), NLR: neutrophil-to-lymphocyte ratio (AUC-0.58), PLR: platelet-to-lymphocyte ratio (AUC-0.4), PMI: platelet mass index (AUC-0.6), RDW: red cell distribution width (AUC-0.58), ROC: receiver operating characteristic, AUC: area under the curve

A total of 200 children were enrolled in the study. An equal number of cases (n = 100) and controls (n = 100) were selected. Table 1 presents the basic characteristics of the participants. The median age of the children was 4.7 (1.0-7.75) and five (2-10) months in the cases and controls, respectively, with 49 (49%) being female in the sepsis group and 47 (47%) in the non-sepsis group. There was no significant difference between the two groups in terms of age and gender. Amongst the sepsis group children, 19 patients had culture-positive sepsis. Median hemoglobin levels were 9.30 (7.97-10.70) g/dL and 11.10 (9.80-12.10) g/dL in the sepsis and non-sepsis groups, respectively. Hemoglobin levels were significantly lower in the sepsis group (p value = 0.01). Median platelet count was significantly lower in the sepsis group (p value = 0.001) than in the non-sepsis group. Median NLR in the sepsis group was 2.53 (1.21-5.53) and in the non-sepsis group it was 2.07 (1.07-3.36) with a significant p value of 0.028. Median PLR was significantly lower in the sepsis group, with a p-value of 0.031. Median PMI in the sepsis group was 130 (94.5-229) fL/nL, and the non-sepsis group was 113.42 (86-156) fL/nL, with a significant p value of 0.01. There was no statistically significant difference in the MPV values and PDW between the two groups. The ROC curves for CRP, RDW, NLR, PLR, and PMI are represented in Figure 1. ROC curve analysis was conducted to evaluate the diagnostic performance of CRP and various hematological markers in predicting clinical outcomes (Table 2). The area under the curve (AUC) values varied across the test variables, indicating differing levels of predictive utility

**Table 1:** Baseline Characteristics of the Participants IQR: interquartile range, NLR: neutrophil-to-lymphocyte ratio, PLR: platelet-to-lymphocyte ratio, MPV: mean platelet volume, PDW: platelet distribution width, PMI: platelet mass index, RDW: red cell distribution width, CRP: C-reactive protein, pSOFA: Pediatric Sequential Organ Failure Assessment, ANC: absolute neutrophil count $ Mann-Whitney U Test *Fisher’s exact test

Parameter	Cases (n=100)	Control (n=100)	P value	Test Statistic	Statistical test
Demographic Data					
Age (years) median (IQR)	3 (1.0-7.25)	5 (2-10)	^$^0.119	5806	Mann-Whitney U Test
Female n (%)	49 (49%)	47 (47%)	^*^0.55	-	Fisher's exact test
Male n (%)	51 (51%)	53 (53%)			
Outcome Data					
Mortality n (%)	32 (32%)	0 (0%)	*0.048	2889	Mann-Whitney U Test
Duration of hospital stay (days) median (IQR)	8.0 (4-15)	7.0 (4-10)	^$^0.008	4146	Mann-Whitney U Test
Use of vasopressors n (%)	61 (61%)	0 (0%)	^*^<0.001	-	Fisher's exact test
Use of oxygen n (%)	98(98%)	35 (35%)	^*^<0.001	-	Fisher's exact test
Use of mechanical ventilation n (%)	55 (55%)	0 (0%)	^*^<0.001	-	Fisher's exact test
pSOFA score median (IQR)	7 (5-12)	1 (0-2)	^*^<0.001	-	Fisher's exact test
Laboratory Markers					
	Median (IQR)	Median (IQR)	p-value		
Hemoglobin (gm/dl)	9.30 (7.97-10.70)	11.10 (9.80-12.10)	^$^0.01	6946	Mann-Whitney U Test
ANC (×10^3^/mm^3^)	9.84 (4.81-16.48)	7.44 (4.57-12.31)	^$^0.062	4205	Mann-Whitney U Test
NLR	2.53 (1.21-5.53)	2.07 (1.07-3.36)	^$^0.028	4099	Mann-Whitney U Test
Platelet Count (×10^3^/mm^3^)	189 (109-410)	346 (270-420)	^$^0.001	6580	Mann-Whitney U Test
PLR	76.11 (34.18-123.7)	91.30 (53.7-141.4)	^$^0.031	5882	Mann-Whitney U Test
MPV (fl)	10.20 (9.30-11.02)	10.10 (9.20-11.10)	^$^0.982	4991	Mann-Whitney U Test
PDW (fl)	11.35 (9.50-14.30)	11.10 (9.30-14.0)	^$^0.722	4854	Mann-Whitney U Test
PMI (fL/nL)	130 (94.5-229)	113.42 (86-156)	^$^0.010	3949	Mann-Whitney U Test
RDW (%)	16.30 (14.70-19.25)	15.50 (14.20-17.50)	^$^0.037	4146	Mann-Whitney U Test
CRP (mg/L)	3.43 (0.84-7.11)	0.41 (0.11-3.27)	^$^0.001	2910	Mann-Whitney U Test

**Table 2:** Analysis of Test Parameters Using ROC Curve NLR: neutrophil-to-lymphocyte ratio, PLR: platelet-to-lymphocyte ratio, PMI: platelet mass index, RDW: red cell distribution width, CRP: C-reactive protein, AUC: area under the curve, CI: confidence interval, PPV: positive predictive value, NPV: negative predictive value, ROC: receiver operating characteristic

Test variable	AUC	Sensitivity (%)	Specificity (%)	Std Error	P value	95% CI	PPV (%)	NPV (%)
NLR	0.58	42	72	0.041	0.032	0.50-0.66	90	23.2
PLR	0.40	82	96	0.041	0.019	0.32-0.48	83.51	1.56
PMI	0.61	25	97	0.040	0.004	0.53-0.69	85.54	24.13
RDW	0.58	63	58	0.041	0.029	0.50-0.66	88.65	20.38
CRP	0.70	77	57	0.037	0	0.62-0.77	89.87	20.33

## Discussion

In this retrospective single-center study, we evaluated the association between CBC parameters and pediatric sepsis using the Phoenix Sepsis Score as a diagnostic tool in a tertiary care center in India. In our study, hemoglobin levels, platelet count, RDW, and indices like the NLR, PLR, and PMI were found to be valuable parameters for the early identification of pediatric sepsis. Given their accessibility, affordability, and routine use in clinical practice, these parameters may serve as valuable adjuncts to clinical assessment, particularly in low-resource settings where advanced diagnostics are limited.

Hemoglobin levels were significantly lower in septic children in our study (p=0.01). This finding is consistent with observations by Bateman et al., who suggested that anemia in sepsis may result from increased vascular permeability, inflammation-mediated suppression of erythropoiesis, or frequent blood sampling during critical care management [18]. Tissue hypoxia caused by anemia can also lead to organ dysfunction, particularly in children with underlying nutritional deficiencies. RDW has been identified as a significant predictor of sepsis, with established associations to increased mortality and prolonged ICU stays [19]. RDW was found to be a significant diagnostic biomarker for pediatric sepsis in our study, with a higher value in the sepsis marker (p=0.04).

Neutrophils play a crucial role in innate immunity and are often mobilized during the early stages of sepsis [20]. The number of neutrophils will rise and the percentage of lymphocytes will fall, depending on the severity of inflammation. Zhong et al. (2021) demonstrated that the NLR significantly correlates with sepsis severity in pediatric patients (p<0.001), with a good predictive value (AUC>0.7) [21]. Tamelytė et al. (2019) reported that NLR was significantly higher in children with sepsis (p<0.006) [22]. Consistent with previous studies, NLR demonstrated significantly higher values in the sepsis group in our study (p=0.03), supporting its potential as a diagnostic tool in pediatric sepsis.

Thrombocytopenia is another well-documented feature of sepsis. Our findings demonstrated significantly lower platelet counts in septic children (p=0.001), consistent with studies by Guclu et al. (2013), which attribute thrombocytopenia in sepsis to enhanced peripheral destruction, splenic sequestration, and bone marrow suppression [19]. In response to increased platelet consumption, the body increases the release of larger, younger platelets, as reflected by an elevated PMI, which was also significantly higher in septic cases (p=0.01) in our study. No published studies have specifically validated the diagnostic utility of PMI in pediatric sepsis, highlighting the novelty and potential clinical relevance of our findings. Platelet indices such as MPV and PDW, although not significantly different in our study, have been proposed in other literature as markers of platelet activation and predictors of sepsis severity. Adem Dursun et al. (2018) reported that MPV values were significantly higher in pediatric patients with sepsis compared to those without sepsis (p<0.007) [23]. In our study, the PLR was found to be statistically significant in association with pediatric sepsis (p=0.03). Several studies, such as by Yin et al. (2025) on neonatal late-onset sepsis (AUC=0.833, p<0.05) and an Indonesian survey done in 2023 on early-onset neonatal sepsis (AUC=0.93, p=0.001), have shown strong diagnostic accuracy of PLR in neonates [24,25]. However, studies evaluating its relevance in the pediatric age group remain scarce. Our findings add to the limited evidence available in the pediatric population, reinforcing the potential role of PLR as a diagnostic marker beyond the neonatal period and highlighting the need for further pediatric-focused investigations. However, though RDW, PMI, PLR, and NLR showed statistically significant p values, their low AUCs limit their use as a standalone diagnostic marker.

One of the notable findings of this study was the significantly longer hospital stay observed in septic patients (p=0.008), underscoring the substantial burden of sepsis on healthcare resources and families. Similarly, Zhong et al. (2021) also reported that patients with severe pediatric sepsis had significantly longer PICU stays (p<0.001), durations of mechanical ventilation (p=0.004), and overall hospitalizations (p<0.001) compared to controls, highlighting the broader impact of disease severity on resource utilization and patient outcomes [21]. Longer duration of hospitalization increases the risk of nosocomial infections, psychological distress for caregivers, and financial hardship-issues particularly relevant in LMICs like India [26].

The primary strength of our study lies in its practical applicability. CBC tests are widely available and inexpensive, making them ideal for integration into sepsis screening protocols in under-resourced settings. Moreover, the case-control design with matched baseline characteristics enhances internal validity. However, several limitations must be acknowledged. The study's single-center design limits its generalizability. The retrospective design is inherently subject to selection bias and potential documentation errors. Additionally, the timing of sample collection was not standardized, which may have influenced biomarker levels. Multivariate analysis, taking into account confounding factors like nutrition status and severity level, was not done. Low AUCs of most parameters limit their potential to be used as individual diagnostic markers in pediatric sepsis.

Future directions should include larger, multicentric prospective studies to validate the diagnostic and prognostic accuracy of CBC-derived indices across diverse pediatric populations. The integration of these parameters into clinical decision-support tools or early warning systems could further enhance sepsis recognition and improve outcomes. Research into combining CBC indices with point-of-care biomarkers, such as CRP or procalcitonin, may offer synergistic value. Based on study findings, these tests can be used as supportive tests in pediatric sepsis rather than being truly diagnostic.

## Conclusions

In conclusion, this study demonstrates that routine CBC parameters - hemoglobin, platelet count, RDW, and derived indices like NLR, PLR, and PMI - show significant associations with early pediatric sepsis. These markers may be used as low-cost, accessible adjuncts to clinical assessment, particularly in settings where rapid, comprehensive diagnostics are not feasible. However, since none of the CBC parameters emerged as a stand-alone diagnostic marker in the current study, therefore they should be used cautiously. Combining multiple hematological parameters may improve predictive accuracy in future clinical models. Further prospective, adequately powered studies taking into account confounding factors like nutritional status and severity of illness are required to validate their role in pediatric sepsis.
